# Near-infrared dye loaded polymeric nanoparticles for cancer imaging and therapy and cellular response after laser-induced heating

**DOI:** 10.3762/bjnano.5.35

**Published:** 2014-03-18

**Authors:** Tingjun Lei, Alicia Fernandez-Fernandez, Romila Manchanda, Yen-Chih Huang, Anthony J McGoron

**Affiliations:** 1 Biomedical Engineering Department, Florida International University, 10555 West Flagler Street, Miami, FL 33174, USA; 2 Cirle, 1951 NW 7th Ave, Suite 13016, Miami, FL, 33136, USA; 3 Physical Therapy Department, Nova Southeastern University, 3200 S. University Dr., Fort Lauderdale, FL 33328, USA; 4 Department of Basic and Applied Sciences, Galgotias University, UP, 201308, India

**Keywords:** hypoxia-inducible factor-1, IR820, nanoparticle, poly(glycerol malate co-dodecanedioate) (PGMD), vascular endothelial growth factor

## Abstract

**Background:** In the past decade, researchers have focused on developing new biomaterials for cancer therapy that combine imaging and therapeutic agents. In our study, we use a new biocompatible and biodegradable polymer, termed poly(glycerol malate co-dodecanedioate) (PGMD), for the synthesis of nanoparticles (NPs) and loading of near-infrared (NIR) dyes. IR820 was chosen for the purpose of imaging and hyperthermia (HT). HT is currently used in clinical trials for cancer therapy in combination with radiotherapy and chemotherapy. One of the potential problems of HT is that it can up-regulate hypoxia-inducible factor-1 (HIF-1) expression and enhance vascular endothelial growth factor (VEGF) secretion.

**Results:** We explored cellular response after rapid, short-term and low thermal dose laser-IR820-PGMD NPs (laser/NPs) induced-heating, and compared it to slow, long-term and high thermal dose heating by a cell incubator. The expression levels of the reactive oxygen species (ROS), HIF-1 and VEGF following the two different modes of heating. The cytotoxicity of NPs after laser/NP HT resulted in higher cell killing compared to incubator HT. The ROS level was highly elevated under incubator HT, but remained at the baseline level under the laser/NP HT. Our results show that elevated ROS expression inside the cells could result in the promotion of HIF-1 expression after incubator induced-HT. The VEGF secretion was also significantly enhanced compared to laser/NP HT, possibly due to the promotion of HIF-1. In vitro cell imaging and in vivo healthy mice imaging showed that IR820-PGMD NPs can be used for optical imaging.

**Conclusion:** IR820-PGMD NPs were developed and used for both imaging and therapy purposes. Rapid and short-term laser/NP HT, with a low thermal dose, does not up-regulate HIF-1 and VEGF expression, whereas slow and long term incubator HT, with a high thermal dose, enhances the expression of both transcription factors.

## Introduction

The synthesis and development of novel polymers and their use for nanoparticle (NP) synthesis has been an important focus of materials science research in the past decade. NPs delivery systems are useful for in vivo applications because their small size (≈100 nm) allows them to escape reticuloendothelial system (RES) uptake, resulting in prolonged plasma circulation times. Moreover, they are able to stabilize and protect their cargo from degradation, including drugs and other types of biomolecules [1–2]. NPs have also proven to be useful in overcoming multidrug resistance (MDR) by preventing the direct interaction of drug exporter pumps with their substrates once encapsulated in NPs [3]. An additional advantage of NPs is that they are passively targeted to tumor sites because of the enhanced permeability and retention (EPR) effect. This effect occurs as a result of a combination of factors, including increased pore sizes of tumor vasculature, fast tumor angiogenesis from increased secretion of vascular endothelial growth factor (VEGF), and poor lymphatic clearance from tumor sites [4]. Because of these advantages, we synthesized a new formulation of polymeric NPs for image-guided therapy based on the polymer poly(glycerol malate co-dodecanedioate) (PGMD) developed in our lab. The work described in this manuscript is based on experiments completed as a partial fulfillment of the requirements for Tingjun Lei’s PhD thesis [5]. Biocompatible and biodegradable PGMD polymers were synthesized through the thermal condensation method by mixing glycol, malic acid and 1,12-dodecanedioic acid (DDA). Following the synthesis of PGMD polymer, PGMD NPs were also successfully formulated.

Optical imaging has several advantages over more traditional imaging techniques (MRI, PET, CT, etc.), such as high spatial resolution, real time imaging, and systems that are usually smaller and less expensive. Near-infrared imaging dyes (wavelength 700–900 nm) are promising for in vivo imaging because light at these wavelengths has minimal absorption by tissue [6–7]. Moreover, some NIR dyes such as indocyanine green (ICG) can be used as both imaging agents and heat generators due to their unique photothermal properties. However, ICG has a plasma half-life of about 3 min and a poor stability in aqueous solution, which complicates the timing of imaging and hyperthermia (HT) [8]. In our previous work, we investigated the commercially available cyanine dye IR820 and proposed that it could be an alternative for ICG. Our studies have shown that IR820 can be used in lieu of ICG in imaging and hyperthermia applications. Three-minute laser exposure (power at 1440 J/cm^2^) with 5 µM IR820 or ICG can elevate the temperature of cell culture media from 37 °C to 42 °C or from 37 °C to 46 °C, respectively [9]. Despite the fact that IR820 has a lower fluorescence yield and results in a lower temperature increase after laser exposure compared to ICG, we have found that either 5 µM IR820 or ICG can be used successfully for in vitro and in vivo optical imaging, and the increased temperature created by IR820 laser exposure is still within the range (usually 41–45 °C) needed for killing cancer cells. More importantly, IR820 has improved in vitro and in vivo stability compared to ICG. The in vitro IR820 degradation half-time is about twice that of ICG. In vivo, the plasma distribution half-life of IR820 is about 15 min, which is 5 times that of ICG; with an elimination half-life of over 30 h for IR820 compared to approximately 2 h for ICG [8]. Based on these advantages we chose IR820 as our near-infrared agent and we synthesized and characterized IR820-PGMD NPs for cancer imaging and HT applications.

HT is used clinically as an adjuvant treatment with chemotherapy and radiotherapy. HT achieves therapeutic benefits by damaging cancer cell proteins and structures as a result of an increase in cell temperature. However, one of the potential problems is that hypoxia-inducible factor-1 (HIF-1) could be up-regulated by HT [10–11]. An overexpression of HIF-1 has often been correlated to a poor therapeutic outcome, since HIF-1 could circumvent the anticancer drug effect by protecting cells from drug-induced apoptosis [12–14]. Moreover, tumor angiogenesis occurs partly by activating the expression of VEGF, which is partially regulated by HIF-1 [15–17]. Given the importance of HIF-1, studies of the effect of HT on this protein are very relevant for therapeutic HT applications in cancer. Goyal et al. and Chandel et al. reported that elevated reactive oxygen species (ROS) levels in cells stabilize HIF-1 expression [18–19]. On the other hand, ROS was also reported to induce mRNA accumulation for heat shock protein 70 (HSP70) [20], which is able to minimize the effect of heat on cells during heat exposure by inducing cells’ thermotolerance [21–22]. Our previous study investigated the effect of HT on cancer cells in a thermal dose-dependent manner, and the results showed that HSP70 was inhibited by indocyanine green (ICG)-induced rapid heating after exposure to laser, so that the thermal protective mechanism of the cells was not initiated [23]. This was compared to the increased expression of HSP70 under slower but longer term heat accumulation by using a cell culture incubator. The results indicated that the promotion of HSP70 was minimized during rapid heating.

As mentioned before, ROS can activate the expression of HSP70. The inhibition of HSP70 during rapid-rate and low thermal dose heating could possibly mean the abolishment of ROS generation, or abolishment of ROS-induced expression of HSP70. It would be important to investigate if laser-IR820-PGMD NP (laser/NP)-induced HT could result in ROS generation and trigger an overexpression of HIF-1. We hypothesized that rapid, short-term and low-dose heat accumulation after laser exposure to IR820-PGMD NPs within cancer cells will not activate ROS production and trigger HIF-1 and VEGF expression. Whereas slow and long-term incubator HT, with high thermal dose, will activate ROS production and result in the promotion of HIF-1 and VEGF expression. The study of cell killing and the cellular response of ROS, HIF-1 and VEGF expression in cancer cells after laser exposure are very important in determining the effect of the heating rate and the amount of thermal dose in the treatment of cancer cells. We used incubator-induced HT to mimic the application of whole-body HT, since the heating process is slow, thus taking a fairly long time to reach the targeted temperature (39–43 °C). Therefore, the comparison between incubator HT and laser/NP HT may provide important information on the effects of different modes of HT used in cancer therapy.

In a previous publication, we described the in vivo pharmacokinetics and biodistribution of IR820-PGMD NPs [24]. The present manuscript concentrates primarily on the in vitro response of cancer cells after hyperthermia. Therefore, this paper focuses not only on the cancer imaging and therapy capabilities of IR820-PGMD NPs, but also on exploring the cellular response following two different HT modes. We first investigated the potential application of IR820-PGMD NPs on cancer imaging and therapy and compared the therapeutic effect to incubator HT. Next, we performed cell-based assays to study ROS, HIF-1 and VEGF expression under these two different heating methods.

## Results

### Characterization of the PGMD polymer and IR820-PGMD NPs

The MW of PGMD polymers measured by GPC column is around 3000 Da. The glass transitional temperature (*T*_g_) is measured to be approximately 42 °C, which is within the range of the IR820 temperature increase after laser exposure. The diameters of void PGMD NPs and IR820-PGMD NPs (see dynamic light scattering (DLS) measurements in Figure S1, Supporting Information File 1) are 90 ± 18.2 nm, and 108 ± 7.4 nm (mean ± SD) respectively. The shape and size of IR820-PGMD NPs were also confirmed with scanning electron microscopy (SEM) imaging (see SEM images in Supporting Information File 1, Figure S2). Polydispersity (PDI) is 0.142 ± 0.007 (mean ± SD), zeta potential is −28.3 ± 6.4 mV (mean ± SD), and the dye loading efficiency is 8.2 ± 0.6 (wt/wt %) (mean ± SD). These results were obtained from ten different NP batches.

### Subcellular localization

**Figure 1:**
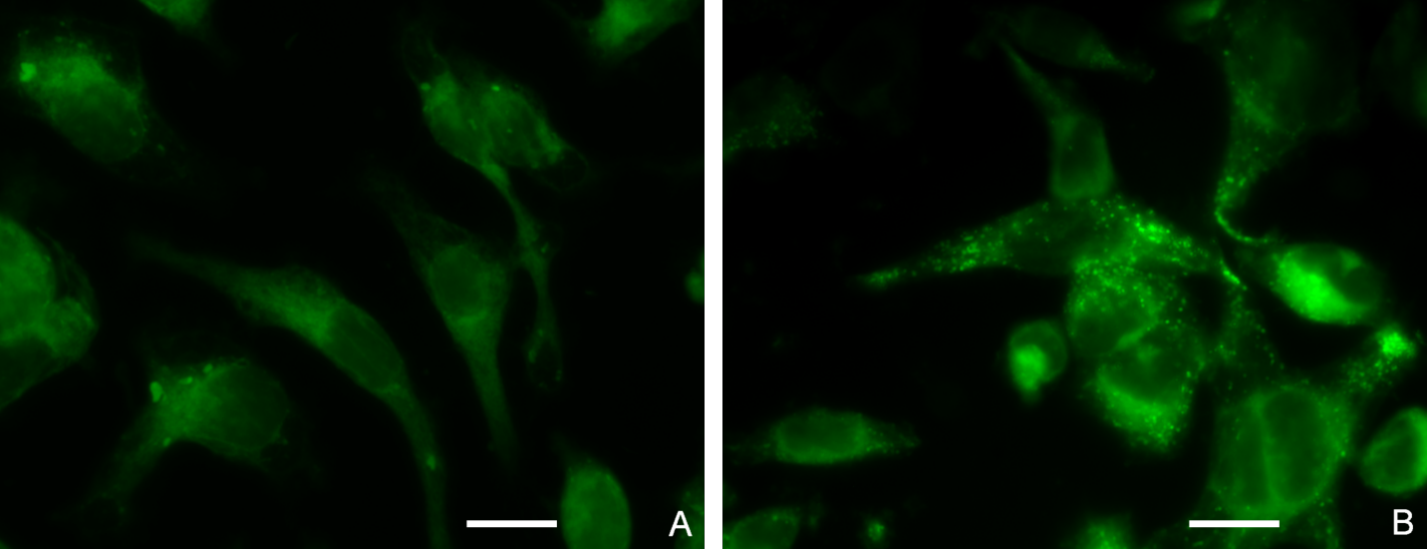
Subcellular localization of free IR820 (A) and IR820-PGMD NPs (B) in SKOV-3 after 24-hour incubation. Scale bar represents 20 µm.

Figure 1 shows images of cells treated with 5 µM free IR820 or 0.05 mg/mL IR820-PGMD NPs (equivalent to 5 µM IR820), and illustrates that the localization of the agents within the cells is similar. Free IR820 is widely spread throughout the cytoplasm, most likely due to interaction with intracellular proteins such as ligandin [25]. In the case of the NP formulation, IR820 released from the NPs should exhibit identical behavior as free IR820, whereas IR820 still within the NPs is expected to be located in endosomes/lysosomes. Lysotracker Blue was used to identify that PGMD NPs were taken up by the cells through an endocytosis pathway (Supporting Information File 1, Figure S5). Calculated image ratio values, *R*, from the fluorescence microscope images show that the NP formulation produces a higher intracellular fluorescence intensity (*R* = 3.75 ± 0.54) (mean ± SD) than the free dye (*R* = 2.89 ± 0.23) (mean ± SD) after 24 h of incubation, although the difference is not statistically significant, possibly due to the small sample size (*n* = 3 for each group).

### HT thermal dose calculation

The temperature curves during 1 h incubator HT and 3 min laser/NP HT are shown in Figure S3 and S4 (Supporting Information File 1). A much slower temperature increase curve was observed in incubator HT compared to the temperature increase in laser/NP exposure. The thermal doses given in these two treatments were calculated according to the CEM_43_ model developed by Sapareto et al. [26] with a slight modification to accommodate for the utilization at 42 °C (CEM_42_) with a smaller empirical value R = 0.25. Laser/NP HT for 3 min with 5 µM IR820-PGMD NPs produced a much lower thermal dose (CEM_42_ = 3.06 min) as compared to the 42 °C incubator HT treatment (CEM_42_ = 25.98 min) over 1 h.

### Cytotoxicity study

**Figure 2:**
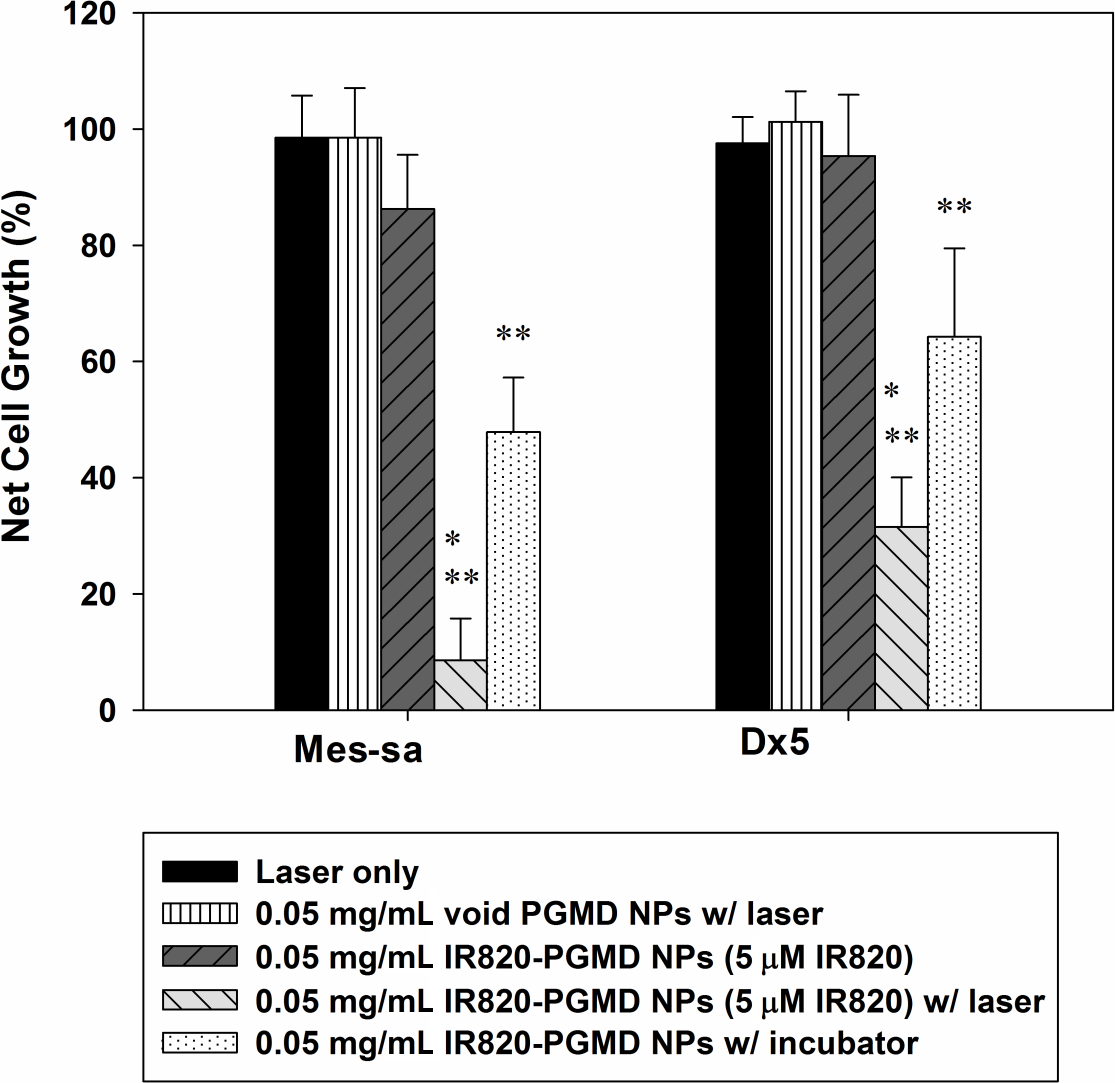
24-hour cytotoxicity profile of IR820-PGMD NPs with laser and incubator exposure in MES-SA and Dx5 cells; *n* = 3, 4 wells/treatment. * *p* < 0.05 (by ANOVA) between laser/NP HT and incubator/NP HT, indicating laser/NP HT results in significantly improved cytotoxicity compared to incubator HT. ** *p* < 0.05 (by ANOVA) between HT and without HT group in both cell lines, indicating significantly higher cancer cell killing was achieved due to HT.

Our group previously described the thermal effects of IR820 in cells exposed to 808 nm laser at a power density of 1440 J/cm^2^. Specifically, exposure to 5 μM IR820 and a 3-minute laser treatment under these conditions produces temperature increases of 5 °C from a baseline of 37 °C [9]. Based on this finding, we used a concentration of 0.05 mg/mL IR820-PGMD NPs (containing approximately 5 μM IR820) in the current study and compared it to the incubator treatment. Figure 2 shows the results of the cytotoxicity study in MES-SA and Dx5. As seen in the figure, laser exposure without concomitant exposure to IR820 did not significantly impact cell growth. It is also noteworthy that NP concentrations equivalent to 5 µM IR820 had a slight cell growth inhibition effect on MES-SA cells. This is in line with our previous observations on the cytotoxicity effects of free IR820 on MES-SA, and seems to be related to the fact that drug-sensitive MES-SA cells are more readily affected by environmental changes and exposure to foreign substances than their drug-resistant counterpart Dx5. Both incubator HT and laser/NP-induced HT killed cancer cells due to the HT effect (*p* < 0.05). Laser/NP HT cause greater cell killing compared to incubator HT (*p* < 0.05), probably because thermotolerance and cell protective mechanisms were not triggered [27].

### ROS production after HT treatment

**Figure 3:**
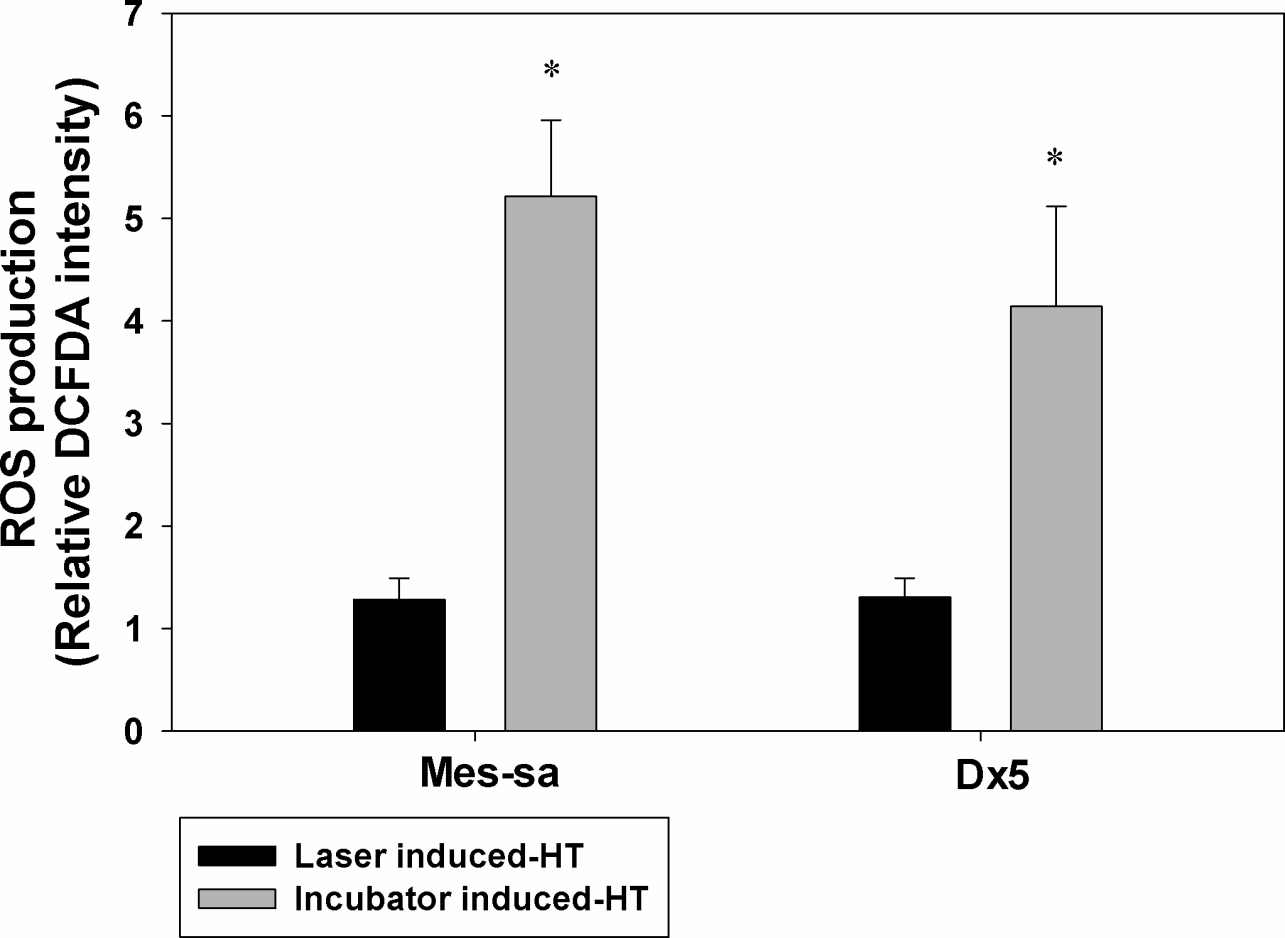
HT-induced ROS production following laser/NPs and incubator was measured in MES-SA and Dx5 cells. Fluorescent dye CM-H2DCFDA was used to measure the fluorescence intensity and normalized to values obtained from the control group (37 °C). *p < 0.05 indicates significant ROS production was observed in incubator induced-HT as compared to control. Laser/NP induced-HT did not result in enhanced ROS production as compared to control. Data presented as mean ± SD, *n* = 3.

ROS production after the two different modes of HT is shown in Figure 3. Incubator HT at 42 °C for 1 h induced production of ROS in both MES-SA and Dx5 cells, whereas ROS production after 3 min of 5 μM laser/NP HT was not different from the control cells that were incubated in a 37 °C incubator probably because much less thermal dose was used, and/or the rapid heating rate does not initiate the ROS production.

### HIF-1 expression

**Figure 4:**
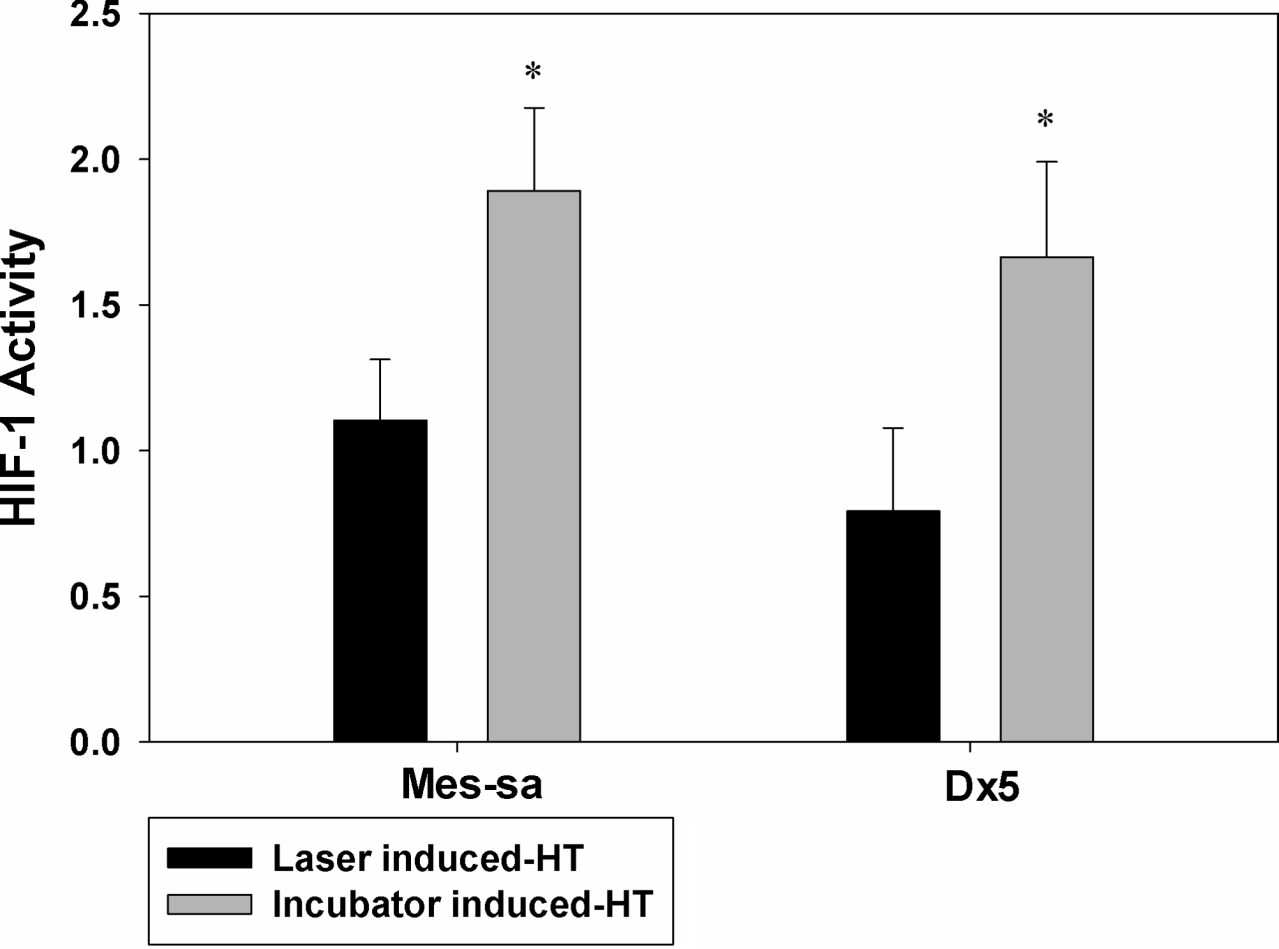
HT-induced HIF-1 expression after laser/NPs and incubator was measured in MES-SA and Dx5 cells. HIF-1 activity was assayed by using HIF-1 ELISA. All measured values were normalized to the mean value of the treatment at 37 °C. * *p* < 0.05 indicates significant HIF-1 expression was observed in incubator induced-HT as compared to control. Laser/NP induced-HT did not result in promoted HIF-1 expression compared to control. Data presented as mean ± SD, *n* = 3.

As expected, incubator HT induced significantly elevated HIF-1 expression as compared to control (*p* < 0.05), while laser/NP HT did not result in significant changes in HIF-1 expression as shown in Figure 4. These results suggest that rapid laser/NP HT did not up-regulate HIF-1 expression either as a result of the rapid heating or low thermal dose or both.

### VEGF expression

**Figure 5:**
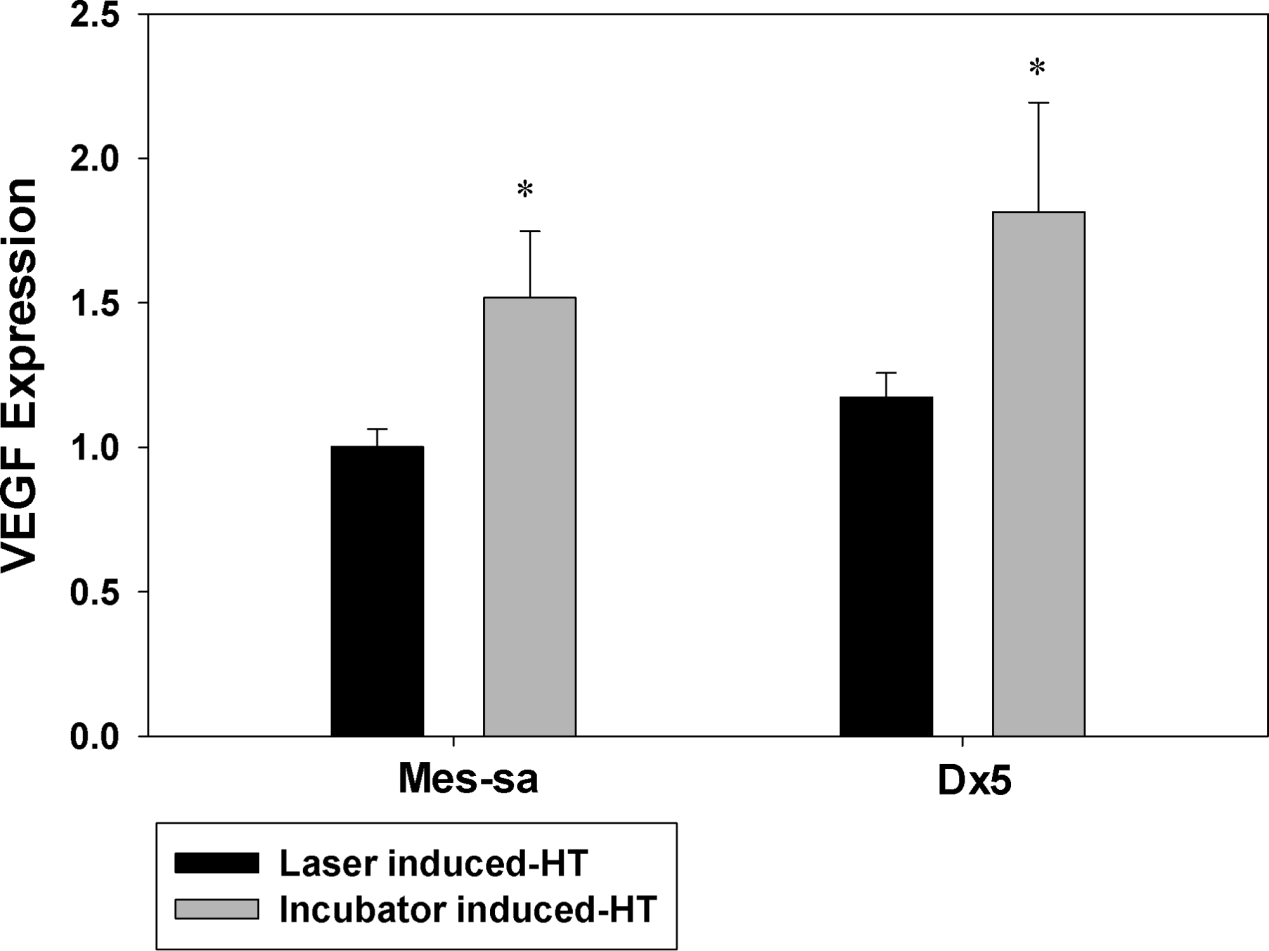
HT-induced VEGF expression after laser/NP and incubator was measured in MES-SA and Dx5 cells. VEGF secretion was measured by using VEGF ELISA. The obtained VEGF expression amount was normalized to SRB value as an indicator of cellular protein amount. All the values measured were then normalized to the controls. * *p* < 0.05 indicates significant VEGF expression was observed in incubator HT as compared to control. Laser/NP HT did not result in enhanced VEGF expression. Data presented as mean ± SD, *n* = 3.

VEGF expression is shown in Figure 5. It is not surprising to observe that VEGF secretion was enhanced after incubator HT, since HIF-1 expression was elevated after incubator HT and VEGF is one of the downstream target genes of HIF-1. Accordingly, we did not observe significant changes in VEGF expression after laser/NP HT, given that laser/NP HT did not have any effect on HIF-1 expression.

### In vivo imaging studies

**Figure 6:**
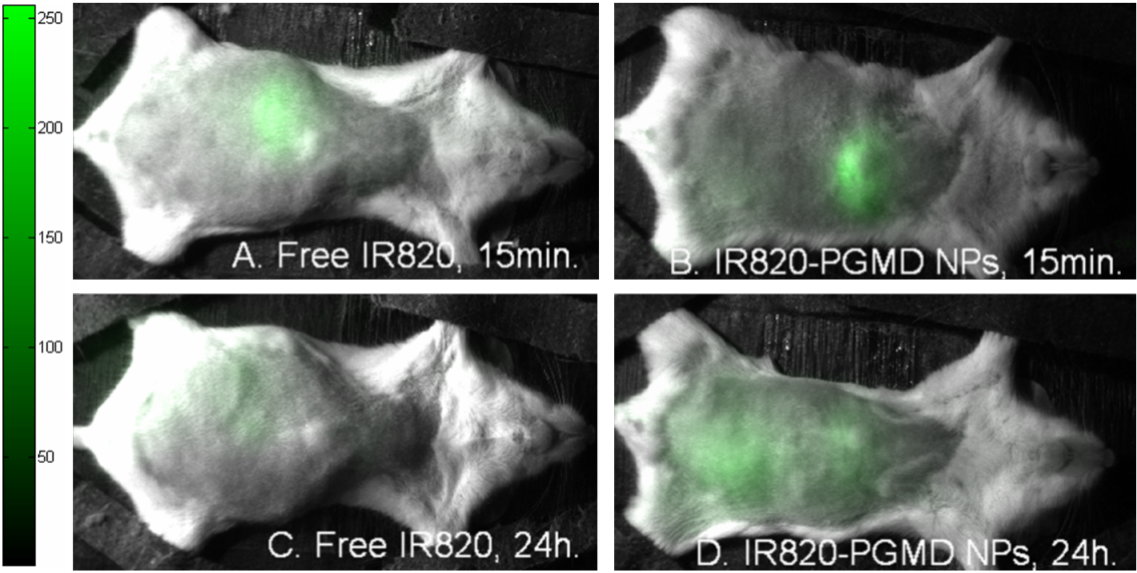
In vivo imaging of free IR820 and IR820-PGMD NPs. Figure 6A and 6B 15 min in vivo imaging. Figure 6C and 6D 24 h in vivo imaging.

In vivo imaging was performed for multiple time points as described in the Experimental section. Images taken at 15 min and 24 h are shown in Figure 6A and Figure 6B, respectively. These images show that the biodistribution of IR820-PGMD NPs is initially very similar to free IR820, as both were processed rapidly through hepatobiliary excretion and start to accumulate in the liver within the first 15 min. After 24 h, it seems that both free dye and NPs were mainly located in the liver. Our previous organ studies showed that considerable IR820 content was also found in the kidneys and the lungs, indicating uptake by RES [24]. However, the IR820 content in kidneys and lungs is lower with NP formulation than in their free form, possibly indicating less RES uptake of NPs, especially in the case of the kidneys. These differences were not statistically significant, probably due to the small sample size and individual variance. The NPs allow for longer image collection times. *R* values show that NPs have significantly higher fluorescence intensity (*R* = 2.37 ± 0.70) (mean ± SD) than does the dye in free form (*R* = 1.42 ± 0.19) (mean ± SD) 24 h after injection (*p* < 0.05). Additionally, our previous pharmacokinetic analysis of plasma samples showed that IR820 plasma concentration 24 h after injection was significantly higher when administered in NP form compared to the free form [24]. Our release kinetics and pharmacokinetics study results [24] seem to indicate that the NP formulation stabilizes IR820, protecting it from degradation and allowing for longer detection windows.

## Discussion

The MW of PGMD polymer is 3000 Da, which is expected for polymers synthesized by polycondensations of these MW monomers of glycerol, malic acid and DDA [24,28]. The size of the IR820-PGMD NPs is around 100 nm, which allows them to escape RES uptake, and as a result, to have reduced plasma clearance rates [2]. The loading of IR820 is equivalent to 5 µM IR820 in 0.05 mg/mL IR820-PGMD NPs, which is sufficient to induce HT. IR820 is amphiphilic and has both hydrophilic and hydrophobic properties, whereas PGMD is hydrophobic. Therefore, there are hydrophobic-hydrophobic interactions between IR820 and PGMD, and IR820 is encapsulated inside the PGMD polymer matrix. Void PGMD NPs do not have any cytotoxicity effect at this concentration. Optical imaging of cancer cells and mice showed that the use of the NP formulation resulted in a stronger fluorescence signal 24 h after injection. This is consistent with the literature reporting that nanoformulations can result in improved plasma circulation time and protect the loading agent from degradation, which would explain the higher intensities observed in vivo when comparing the NP form with the free dye [29–30]. Our pharmacokinetics study showed that IR820-PGMD NPs administration results in significantly increased IR820 plasma concentration 24 h after injection compared to free IR820. In addition, our biodistribution studies showed that kidney IR820 dye content was lower in NP form than in free IR820 form, which means less IR820 was excreted through the renal system when in NP form. This is consistent with kidney excretion being limited to very small particles and small molecules.

The cytotoxicity studies showed that laser/NP induced HT caused significantly higher cell killing than incubator HT, although a much lower thermal dose was given to the cells. In the commonly used CEM_43_ model for thermal dose calculation, which normalizes the thermal dose to cumulative equivalent minutes at 43 °C [26], the temperature and the duration of heating can be used to define thermal damage. Our previous paper and other groups’ reports indicate that the rate of photothermal treatment might also affect the HT outcome, because under rapid heating the cells are not able to initiate protective mechanisms by inducing the expression of proteins of the heat shock family to reduce DNA damage [23,31]. Although the laser/NP HT produced approximately 9 times less thermal dose than incubator HT, it still resulted in significantly higher cytotoxicity than incubator HT, thus confirming the importance of the heating rate. Note that the final temperature reached in both modes of HT was identical.

Madamanchi’s group reported that ROS can up-regulate HSP70 protein levels by binding signal transducers and activators of transcription (STATs) to the HSP70 promoters in vascular smooth muscle cells (VSMCs) [32]. This group exposed VSMCs to H_2_O_2_ and found that the cytoplasmic janus tyrosine kinase 2 (JAK2)/STAT pathway can up-regulate HSP70 and minimize oxidative stress effects on the cells. The inhibition of HSP70 expression under laser/NP HT probably means no enhancement of ROS production within the cells. Our ROS detection experiments support this hypothesis, showing that no significant ROS was produced inside the cells after laser/NP HT compared to controls. However, when incubator HT was used to mimic conditions more similar to whole body HT, we observed significant intracellular ROS production. This result is consistent with Moon et al. reporting that ROS was activated when a slow water bath HT was applied to cells. HT can activate the ERK pathway and increase NADPH oxidase activity, which leads to the production of ROS [10]. Based on our results, it seems that the application of rapid laser/NP HT to cells will not induce an increase of ROS. However, the specific mechanism of ROS abolishment within cells after laser/NP HT has to be studied further.

Following the inhibition of ROS production in laser/NP HT treatment, we did not observe enhanced HIF-1 expression. However, HIF-1 up-regulation was observed in slow and longer term HT, probably because ROS production was activated in the heating process. Other groups have also suggested that the presence of ROS is able to up-regulate HIF-1 expression [18,33]. HIF-1 is very important as a therapeutic target [34]. Traditional HT with slow and long-term heating appears beneficial as an adjuvant therapy for radiotherapy and chemotherapy since it can hinder DNA damage repair mechanisms and increase drug delivery by enhancing its diffusion into the tumor [35–36]. However, this heating modality is also able to induce up-regulation of HIF-1, and the overexpression of HIF-1 could compromise the therapeutic effect by increasing drug resistance by an up-regulation of p-glycoprotein and by reducing cancer cells drug senescence [37–38]. Our results showed that VEGF secretion was also elevated along with the up-regulation of HIF-1, which could potentially result in enhanced tumor angiogenesis. The combination of HT and other therapies could elevate the HIF-1 expression to an even higher extent than single therapy, which could alter tumor cell behavior and promote the aggression of cells. Therefore, it is important to review the possible molecular effects of HT in considering its application as an adjuvant therapy, as other groups have reported that HIF-1 can also be up-regulated by radiotherapy and chemotherapy [39–41]. Based on our study, IR820-PGMD NPs could be used for HT applications without inducing the adverse effects of HIF-1. The HT therapeutic effect might be determined more by the temperature and the heating rate and perhaps less by the total thermal dose. Due to the usage of laser/NP HT we did not observe enhancement of HIF-1 and VEGF expression, but an improved therapeutic outcome was still achieved compared to incubator HT. Despite these promising results for laser/NP HT, further studies have to be performed to determine treatment parameters, such as how to efficiently deliver these NPs and the timing for HT treatment.

## Conclusion

In summary, we successfully developed IR820-PGMD NPs, which are promising as theranostic agents with multifunctional imaging and HT capabilities. These NPs, when tested in vitro and in vivo, are able to yield higher fluorescence intensity than free IR820 24 h after incubation or 24 h after i.v. injection of equivalent dye concentrations, allowing for longer imaging collection times and potentially widening the window for HT applications. We also proved in our study that the use of IR820-PGMD NPs and laser/NP HT will neither activate ROS expression, nor induce HIF-1 and VEGF expression, which could yield a beneficial therapeutic outcome. This study is an extension of the current knowledge of delivery of HT in NP form, and we believe it will have a significant impact on the application of nanotechnology on cancer imaging and therapy.

## Experimental

### Chemicals and cell-based assays

The following materials were purchased from Sigma-Aldrich (St. Louis, MI): Malic acid, 1,12-dodecanedioic acid (DDA), dimethylsulfoxide (DMSO > 99.9%, reagent grade), pluronic F-127, Dulbecco phosphate-buffered saline (DPBS), phosphate buffered saline (PBS), IR820, penicillin-streptomycin solution, tetrahydrofuran (THF) and trypsin-EDTA. Glycerol was purchased from MP Biomedical (MP Biomedical, LLC, Solon OH). 5-(and-6)-chloromethyl-2′,7′-dichlorodihydrofluorescein diacetate, acetyl ester (CM-H2DCFDA) was purchased from Invitrogen, (Invitrogen, NY), human/mouse total HIF-1 alpha cell-based ELISA and human VEGF quantikine ELISA kit were purchased from R & D systems (R & D Systems, MN).

### Synthesis and characterization of PGMD polymer

This procedure has been previously described by our group [24]. Briefly, a mixture of glycerol, DDA and malic acid (7:3 DDA:malic acid; 1:1 glycerol:DDA/malic acid) was heated to 120 °C for 48 h. Malic acid allows us to control the degree of hydrophilicity and in turn the glass transition temperature (*T*_g_). Characterization was performed by differential scanning calorimetry (for glass transition temperature) and gel permeation chromatography (for molecular weight, based on a calibration curve of polysterene standards).

### Synthesis and characterization of IR820-PGMD NPs

IR820-PGMD NPs were prepared by using an oil-in-water emulsification solvent evaporation technique followed by centrifugation at 5000 rpm for 5 min, and dialysis at MWCO 1000 Da to remove any free IR820 residue. After preparation, the particles were freeze-dried and lyophilized for 48 h. To measure the IR820-PGMD NP size distribution, 100 µL IR820-PGMD NPs were resuspended in 3 mL deionized (DI) water. Then, the solution was measured for average size, size distribution plot based on intensity plot, polydispersity, and zeta potential with a Malvern Zetasizer (Malvern Instruments, Worcestershire, United Kingdom). The size of the particles was measured by determining a correlation function and fitting a polynomial to the correlation function. We used the cumulant analysis as a fitting model for the correlation function in our study. The average particle size, polydispersity, and zeta potential were determined from 10 different NP batches. The DLS intensity plot was obtained from one batch of void PGMD NPs and IR820-PGMD NPs. Scannning Electron Microscopy (SEM, JEOL-JEM) was also used to characterize the NPs shape and size. The loading of IR820 in NP's was evaluated by using a Cary WinUV spectrophotometer (Varian/Agilent Technologies, Switzerland).

### In vitro studies of NPs

Cancer cells MES-SA, Dx5, and SKOV3 were purchased from American Type Culture Collection (Manassas, VA) along with McCoy’s 5A medium and fetal bovine serum. Cell culture supplies were purchased from Fisher Scientific (Pittsburg, PA), and penicillin was purchased from Sigma-Aldrich. Cell culture conditions were as described in our previous publication [24], with 1% penicillin and 10% fetal bovine serum supplementation.

### Subcellular localization of the NPs

SKOV-3 cells were plated in a 24-well tissue culture plate at densities of 4 × 10^4^ cells per well. After overnight incubation to allow for attachment and confluence, we replaced the medium with the test solutions, namely 5 µM free IR820 or 0.05 mg/mL IR820-PGMD NPs (equivalent to 5 µM IR820). Plates were kept at physiological temperature in the dark inside an incubator. Subcellular localization of the IR820-PGMD NPs was identified by incubating 5 µM Lysotracker Blue (Invitrogen, NY) with cells for 10 min at the end of the experiment, followed by 3 × wash with PBS, and fixation with 4% (vol/vol) formaldehyde. Fluorescence images were obtained by using a 60 × water merged objective and a CCD camera, with fluorescence filters of λ_ex_ = 775 nm, λ_em_ = 845 nm for IR820, and λ_ex_ = 355 nm, λ_em_ = 420 nm for LysoTracker Blue. After processing to add pseudo color (IPLab, Qimaging, Canada), the images were imported into Matlab (MathWorks, Massachusetts) and analyzed to determine the intensity ratio *R*. First, the intensity of each pixel was background-subtracted, and the region of interest was defined as being composed of any pixels with above-background intensity values (defined as an intensity of at least 2 out of a 255 scale after background subtraction). The ratio *R* was then determined by normalizing the total pixel intensity of this region of interest to its total area.

### HT treatment

Two different heating modes, namely (1) an incubator and (2) a laser/NP HT delivery system, were used for in vitro studies. Detailed descriptions of the heating systems and the temperature calibration for both heating modes were provided in our previous paper [23]. Note that when incubator HT was used, cells were incubated with the same concentration of IR820-PGMD NPs as used in laser/NP HT in order to eliminate the effect of NPs by themselves.

### Cytotoxicity assessment

Cell viability after five different treatments (laser only, void PGMD NPs w/ laser, IR820-PGMD NPs, IR820-PGMD NPs w/ laser, incubator HT w/ IR820-PGMD NPs) was measured with the Sulforhodamine B colorimetric (SRB) assay 24 h post-treatment, as previously described in our publications [24,42]. The effect of each treatment on cell growth was normalized to the growth of the control group, which did not receive any treatment.

### Cell-based assays for the detection of ROS, HIF-1 and VEGF expression

#### Study of ROS expression

Intracellular ROS level was measured by using the fluorescent dye CM-H2DCFDA, which is converted into a nonfluorescent derivative (H2DCF) by cellular esterases after uptake by cells. Then, H2DCF can be oxidized to highly fluorescent 2′,7′-dichlorofluorescein (DCF) in the presence of ROS. After HT (either 1 h incubator HT or 3 min laser HT), cells were washed with PBS and collected by incubating with trypsin for 5 min. The same number of cells were counted and incubated with CM-H2DCFDA in the dark. After 30 min, cells were briefly washed with PBS, and the intensity of DCF was measured by a flow cytometer (BD Accuri C6, NJ).

#### Study of HIF-1 expression

To investigate HIF-1 expression in both incubator HT and laser/NP HT, a human/mouse enzyme-linked immunosorbent assay (ELISA) was used to detect the expression of HIF-1 by using specific HIF-1 antigen. Basal level HIF-1 expression was identified in cells incubated at normal temperature (37°C). HIF-1 expression was measured immediately after HT by reading the plate with a fluorescence plate reader (GENios, TECAN, CA) with an excitation at 540 nm and an emission at 600 nm to measure the amount of total HIF-1 in the cells. Then, the plate was read with an excitation at 360 nm and an emission at 450 nm to measure the amount of total cytochrome c in the cells. Finally, the HIF-1 amount was normalized to the amount of cytochrome c and expressed as HIF-1 activity.

#### Study of VEGF expression

Cancer cell culture medium was collected 6 h after HT. After centrifuging cell culture media for 10 min at 14000 rpm, 200 µL of supernatant was added into a 96-well plate provided in a human quantikine VEGF ELISA kit. VEGF levels were quantified following the kit protocol, and a sulforhodamine B (SRB) assay (Invitrogen, NY) was used to determine the amount of cellular protein in each well. Subsequently, the measured VEGF amount was normalized to SRB value and the calculated results were normalized to controls.

### In vivo optical imaging

Animal studies were performed following the regulations of the Institutional Animal Care and Use Committee. Twenty-four Nd4 Swiss Webster mice (25–30 grams, 9 weeks old) were purchased from Harlan (Indianapolis, IN), and randomly distributed into 8 different experimental groups based on two factors: time elapsed between injection and data collection (15 min, 30 min, 60 min, and 24 h), and solution injected (0.2 mL of either free IR820 or IR820-PGMD NPs in PBS). Injected solution concentration was matched to an IR820 dose of 0.24 mg/kg of body weight [43]. The in vivo biodistribution of the NPs was recorded with a CCD camera (Qimaging, Canada) coupled with a NIR filter (λ_ex_ = 785 nm, λ_em_ = 820 nm). Later, the images were processed with Matlab to calculate the image fluorescence intensity ratio *R* as described above.

### Statistical analysis

Statistically significant (*p* < 0.05) differences in responses between the treatment groups and control groups was analyzed by ANOVA or t-test (SPSS, Chicago, Illinois).

## Supporting Information

File 1 Additional experimental details.
