# Supplementary material for: Near-infrared dye loaded polymeric nanoparticles for cancer imaging and therapy and cellular response after laser-induced heating
Source: Beilstein J Nanotechnol. 2014 Mar 18;5:313–22. doi: 10.3762/bjnano.5.35 (PMC3999826; doi:10.3762/bjnano.5.35)
Supplement: File 1 — Additional experimental details. [file Beilstein_J_Nanotechnol-05-313-s001.pdf]

Supporting Information

for

Near-infrared dye loaded polymeric nanoparticles for cancer imaging and therapy and cellular response after laser-induced heating

Tingjun Lei[1,2], Alicia Fernandez-Fernandez[1,3], Romila Manchanda[1,4], Yen-Chih Huang[1] and Anthony J. McGoron*[1]

Address: [1]Biomedical Engineering Department, Florida International University, 10555 West Flagler Street, Miami, FL 33174, USA and [2]Cirle, 1951 NW 7th Ave, Suite 13016, Miami, FL, 33136, USA, [3]Physical Therapy Department, Nova Southeastern University, 3200 S. University Dr., Fort Lauderdale, FL 33328, USA and [4]Department of Basic and Applied Sciences, Galgotias University, UP, 201308, India.

Email: Anthony J. McGoron. - mcgorona@fiu.edu

*Corresponding author

Additional experimental details

Dye loading

IR820-PGMD NPs were dissolved in dimethyl sulfoxide (DMSO), and the absorption spectrum of the samples was measured following serial dilutions. The maximum peak intensities were corrected with DMSO blank subtraction, plotted, and fitted to a linear model. The concentration of IR820 in the NPs was determined using a standard calibration curve of IR820 in DMSO.

Dynamic light scattering (DLS) measurement

As described in the experimental section in the manuscript, the DLS intensity plots were obtained from one batch of void PGMD NPs and one batch of IR820-PGMD NPs.

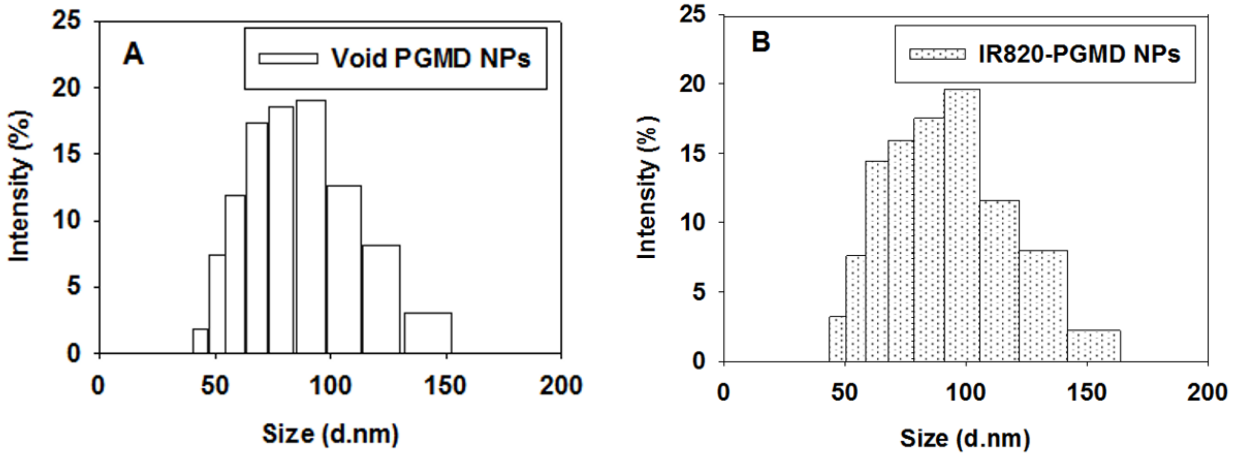

Figure S1: DLS measurement of void PGMD NPs and IR820-PGMD NPs.

Scanning electron microscopy (SEM) imaging

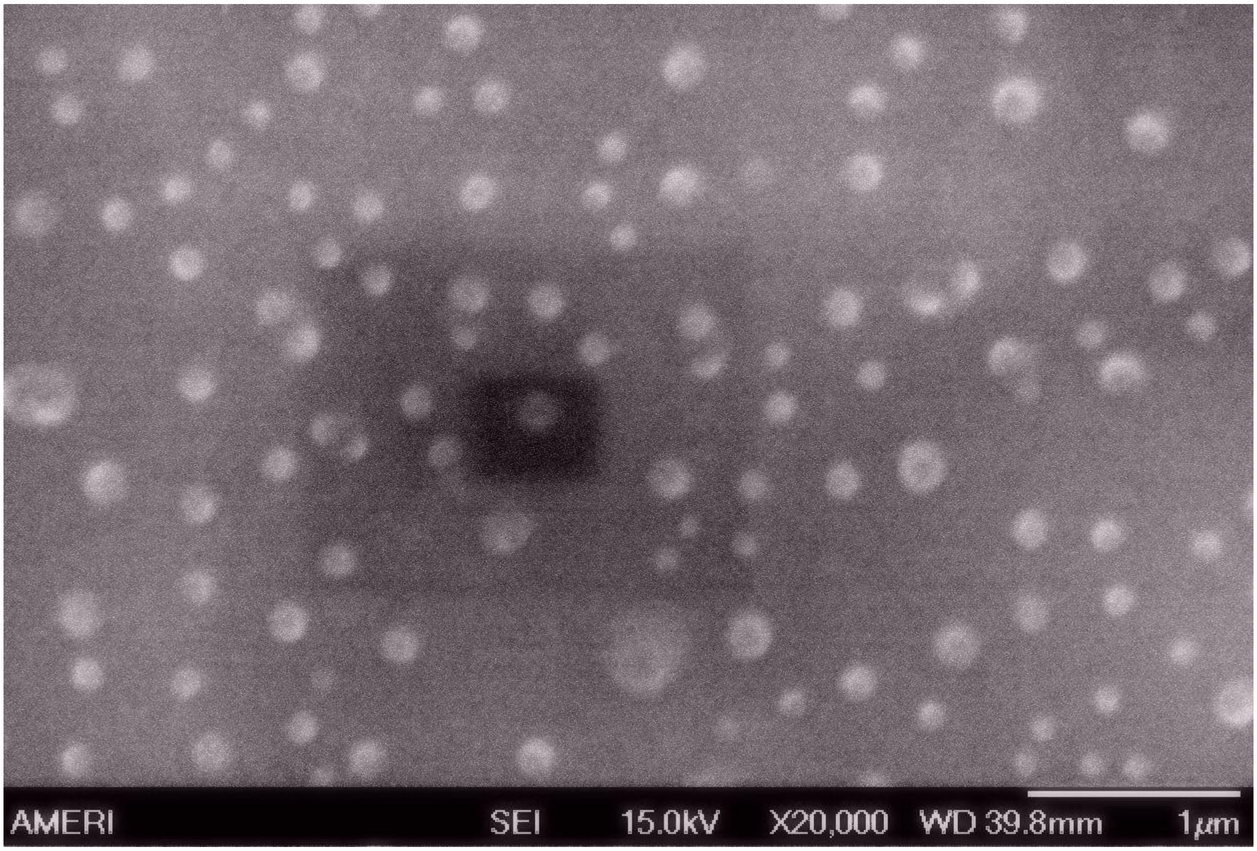

Figure S2: SEM images of IR820-PGMD NPs.

Heating modality

1. Incubator hyperthermia (HT) delivery system and temperature increase profile

In an attempt to mimic conventional whole body HT, a Hera incubator was used as the energy source. The temperature of the incubator was set to approximately 42°C. A ninety-six-well plate, which was originally incubated in the 37°C incubator, was transferred to the 42°C incubator. A temperature calibration study was carried out to determine the temperature profile of the transferred 96-well plates, shown in Figure S3.

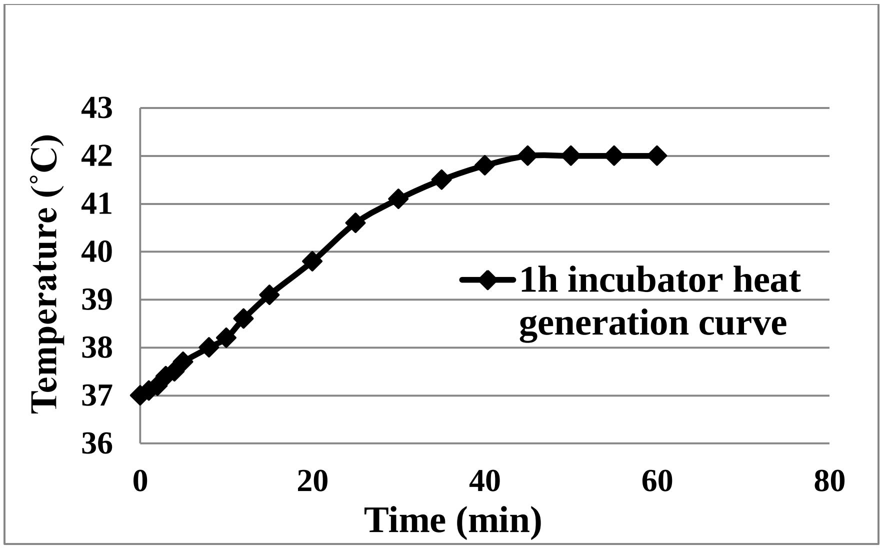

Figure S3: Temperature profile during 1 hour incubator HT.

2. Laser-IR820-PGMD NP HT delivery system and temperature increase profile

In order to deliver rapid HT from within the cell, IR820 has to be first taken up by cells and then activated by a NIR laser. For this purpose, a laser heating system was designed. The system is comprised of a laser module (RLDH808-1200-5, Roithner Lasertechnik, Austria), a laser holder, a heated stage insert (WPI Heated Stage Insert, World Precision Instruments Inc, Sarasota) and a mobile stage with an extension arm. The whole system is enclosed within a box to ensure operator safety and minimize effects of air currents. The heated stage insert, placed on a mobile stage positioned directly below the laser, was powered by an external source to ensure that the plate is at 37 °C prior to the laser application.

The NIR laser source emits light at 808 nm with an output power of 1.2 Watts. Given its 15 mm^2 spot size, the calculated power density is 1440 J/cm^2. This small spot size also guarantees that

only one well is excited at a time. The exact positioning of a well with the laser beam was achieved by moving the stage with an extension arm located outside of the box. The arm was used to move the location of the well plates, thereby allowing different wells to be exposed to the NIR energy one at a time without opening the box. The experimental setup does not use any optical filters or optical lenses to focus the square beam.

The temperature profile of IR820-PGMD NPs at different concentrations after 3-minute laser exposure is shown in Figure S4. After 3-min laser exposure, the temperature of IR820 decreased due to IR820 photobleaching.

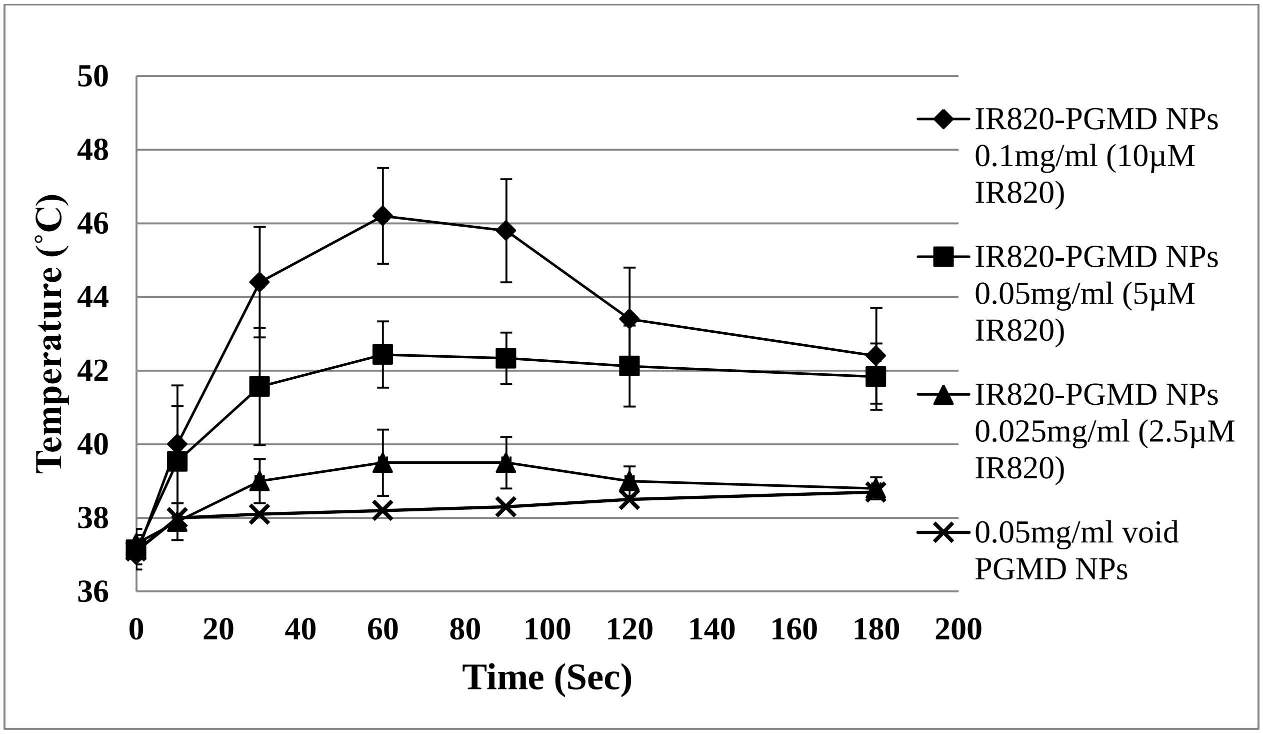

Figure S4: Temperature profile during 3-minute laser heating with different concentrations of IR820-PGMD NPs. Experiments were repeated 3 times.

3. Thermal isoeffective dose

The term "thermal isoeffective dose" is used to compare different time-temperature combinations which produce the same cell killing effect. This method was used since the cell death rate under HT is exponentially related to both time and temperature. The relationship of time and temperature can be expressed mathematically by the following isoeffect equation:

$$t_1 = t_2 * \mathrm{R}^{T_1 - T_2} \quad (1)$$

where t_1 and t_2 are the duration of treatment at temperature T_1 and $T_{2,}$ respectively. Originally, R is a function of temperature, however, an estimation of R as a constant will give an error of less than 2% in the temperature between 37 °C to 46 °C described by Sapareto et al. [1]. Hence, R is assumed to be 0.5 above 42 °C and 0.25 below 43 °C [1]. Based on Equation 1, different thermal doses can be converted to the cumulative equivalent minutes at 43 °C (CEM_{43}). From Equation 1, we did a slight modification and set T_1 to be 42 °C and T_2 to vary during heat treatment, so that we obtain Equation 2. The integral upper limit t is the end time of the experiment (3 minutes or 60 minutes).

$$CEM_{42} = \int_0^t \mathrm{R}^{42 - T(t)} dt \quad (2)$$

Colocalization of IR820-PGMD NPs and LysoTracker Blue fluorescence staining dye

Subcellular localization images are shown in Figure S5. We used Lysotracker Blue to stain SKOV-3 lysosomes. Figure S5A shows the staining of Lysotracker Blue to the lysosomes in the blue fluorescence channel. Whereas, Figure S5B shows the fluorescence of IR820 (red), and

Figure S5C is the overlay image, which shows the co-localization of IR820-PGMD NPs and lysosomes, indicating that PGMD NPs were probably taken up into cells by endocytosis.

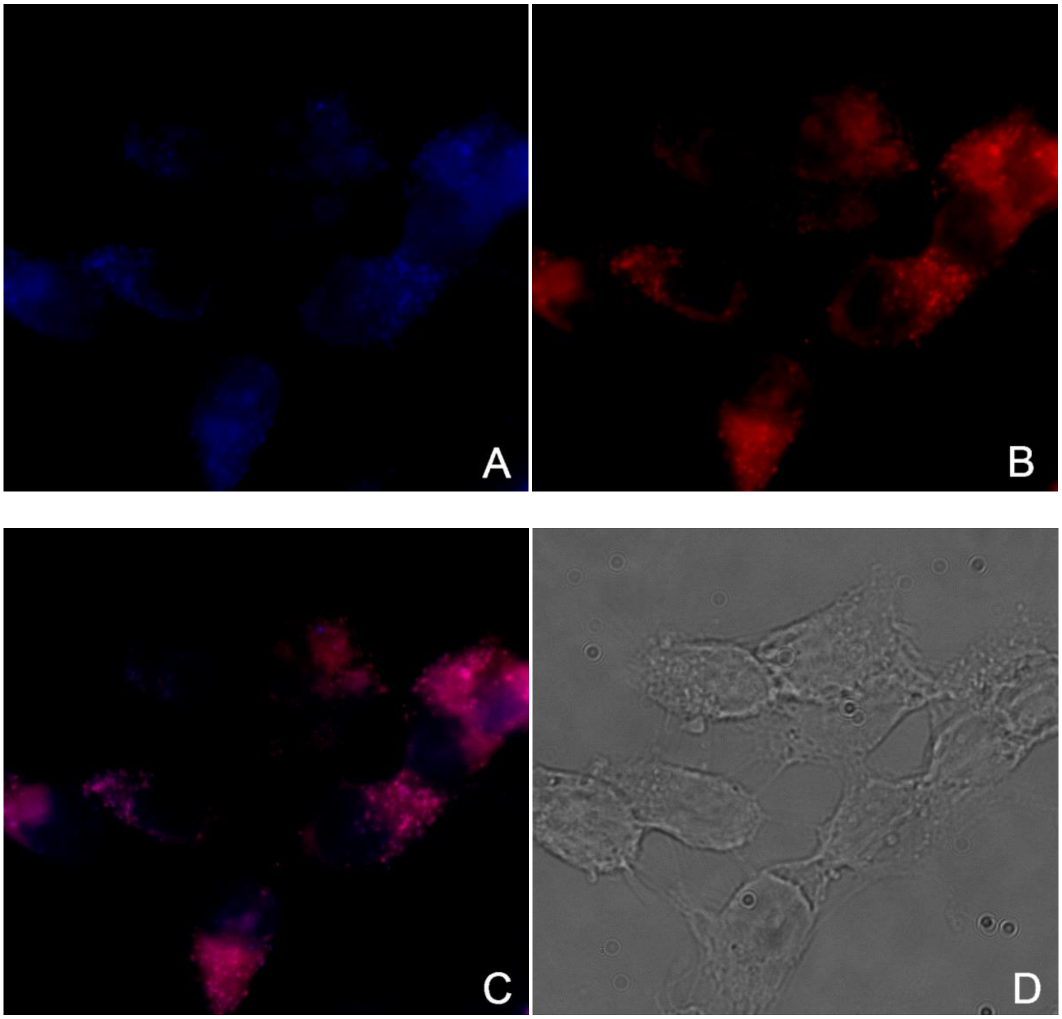

Figure S5: Subcellular localization of IR820-PGMD NPs in SKOV-3. All the images were taken after 24 hours incubation of NPs with cells and were merged with pseudo color by the software (IPLab, Qimaging). A. Lysotracker Blue fluorescence; B. IR820 fluorescence of IR820-PGMD NPs; C. merged picture of A and B. D. Phase contrast image.

Reference

1. Sapareto, S. A.; Dewey, W. C. *Int. J. Radiat. Oncol., Biol., Phys.* **1984,** *10,* 787–800. doi:10.1016/0360-3016(84)90379-1
